# A proof-of-concept study of ultrasound-guided continuous parasacral ischial plane block for postoperative pain control in patients undergoing total knee arthroplasty

**DOI:** 10.1186/s13018-024-04822-9

**Published:** 2024-06-08

**Authors:** Peng Ye, Ting Zheng, Cansheng Gong, Xuan Pan, Zhibin Huang, Daoyi Lin, Xiangyan Jin, Chunying Zheng, Xiaochun Zheng

**Affiliations:** 1 Department of Anaesthesiology, Shengli Clinical Medical College of Fujian Medical University, Fujian Provincial Hospital, Fuzhou, China; 2 Fujian Provincial Key Laboratory of Emergency Medicine, Fujian Provincial Key Laboratory of Critical Care Medicine, Fujian Provincial Co-Constructed Laboratory of “Belt and Road,”, Fujian Emergency Medical Centre, Fuzhou, China

**Keywords:** Parasacral ischial plane block, Postoperative analgesia, Sacral plexus block, Ultrasound-guided continuous nerve blocks, Oxycodone

## Abstract

**Background:**

Continuous peripheral nerve blocks are widely used for anesthesia and postoperative analgesia in lower limb surgeries. The authors aimed to develop a novel continuous sacral plexus block procedure for analgesia during total knee arthroplasty.

**Methods:**

The study comprised two stages. In Stage I, the authors built upon previous theories and technological innovations to develop a novel continuous sacral plexus block method, ultrasound-guided continuous parasacral ischial plane block (UGCPIPB) and subsequently conducted a proof-of-concept study to assess its effectiveness and feasibility. Stage II involved a historical control study to compare clinical outcomes between patients undergoing this new procedure and those receiving the conventional procedure.

**Results:**

The study observed a 90% success rate in catheter placement. On postoperative day (POD) 1, POD2, and POD3, the median visual analog scale (VAS) scores were 3 (range, 1.5–3.5), 2.5 (1.6–3.2), and 2.7 (1.3–3.4), respectively. Furthermore, 96.3% of the catheters remained in place until POD3, as confirmed by ultrasound. The study revealed a significant increase in skin temperature and peak systolic velocity of the anterior tibial artery on the blocked side compared with those on the non-blocked side. Complications included catheter clogging in one patient and leakage at the insertion site in two patients. In Stage II, the novel technique was found to be more successful than conventional techniques, with a lower catheter displacement rate than the conventional procedure for continuous sciatic nerve block.

**Conclusion:**

UGCPIPB proved to be an effective procedure and safe for analgesia in total knee arthroplasty.

**Chinese Clinical Trial Registry Number:**

ChiCTR2300068902.

**Supplementary Information:**

The online version contains supplementary material available at 10.1186/s13018-024-04822-9.

## Background

The continuous peripheral nerve block technique (CPNB) has been widely used in lower limb surgery for anesthesia and postoperative analgesia. CPNB allows individualized adjustments of doses and concentrations of local anesthetic to achieve optimal analgesia compared to single injection blocks [1]; therefore, CPNB is more suitable for postoperative analgesia [1–4]. However, catheter placement for CPNB is technically challenging. Specifically, the needle and catheter must often be close to the nerve to ensure effective optimal analgesia, increasing the risks of nerve irritation and damage [4–6].

CPNB for total knee arthroplasty mostly focuses on the safe and easily operable superficial nerves or fascial planes, including the femoral nerve, adductor canal, and fascia iliaca compartment block (FICB). Conversely, for deeper nerves such as the sacral plexus, CPNB is rarely used because the sacral plexus is located on the belly side of the piriformis, surrounded by complex anatomical structures such as the colon and superior gluteal artery. Hindered by the current level of ultrasound image resolution and obstruction of the ischium, it is often difficult to identify the sacral plexus. Thus, implementation of the continuous sacral plexus block is challenging.

The sacral plexus and its branches play a major role in the innervation of the posterior knee joint capsule [7].However, the analgesic treatment of the posterior knee joint capsule is often not given adequate attention. Therefore, identifying the fascial plane where the sacral plexus is located to perform nerve blocks or catheter insertion instead of administering local anesthetics directly around deep nerves can reduce the risk of nerve injury and address the deficiency of incomplete analgesia in lower limb surgeries.

Venkataraju et al. proposed the theory of parasacral ischial plane block (PIPB) and transformed the conventional sacral plexus block into a fascial plane block [8]. Sacral plexus blocks can be performed instead of administering local anesthetics directly around deep nerves. The potential fascial interspace also enables the placement of a catheter in the parasacral fascial plane. Therefore, if a catheter can be placed in the plane of the parasacral ischial plane, analgesia can be provided in the posterior thigh, knee, lateral crural, and foot, and when combined with fascia iliaca compartment or lumbar plexus blocks, analgesia can be provided in most areas of the lower extremity. Prior to the application of a new analgesic technique in total knee arthroplasty, it may be necessary to conduct a feasibility study using a single-arm small sample. In this proof-of-concept study, we aimed to develop a new continuous sacral plexus block procedure using ultrasound-guided continuous parasacral ischial plane block (UGCPIPB) and to verify its efficacy and safety.

## Methods and methods

### Study design

**Fig. 1:**
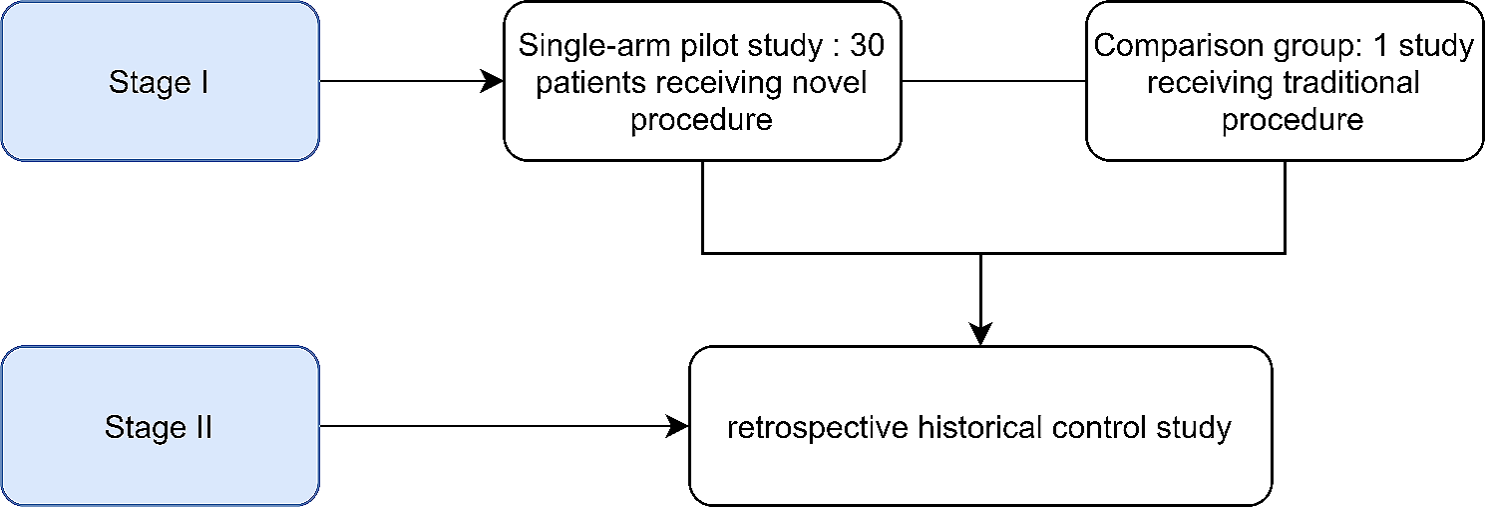
Flowchart of the study

This prospective, single-arm, proof-of-concept study was approved by the Ethics Committee of Fujian Provincial Hospital, China (approval number: K2023-02-003) on February 9, 2023, before patient recruitment and sample collection. Written informed consent was obtained, and the study was registered in the Chinese Clinical Trial Registry (ChiCTR2300068902) before recruitment on March 1, 2023. The first patient enrolled was on March 7, 2023. This study comprised two stages. In Stage I, the authors developed a novel continuous sacral plexus block method, UGCPIPB.Subsequently, to verify the safety and efficacy of UGCPIPB, the authors performed a single-arm proof-of-concept study with this procedure in patients undergoing lower limb surgery. In Stage II, the authors conducted a retrospective historical control study to further assess the efficacy and safety of the new procedure. The flowchart of the study design is presented in Fig. 1.

### Stage I

#### Study population

The authors aimed to recruit 30 patients. The inclusion criteria were as follows: age 18–70 years; ASA I–II level; body mass index (BMI) of 18–30 kg.m^− 2^; and scheduled elective total knee arthroplasty. The exclusion criteria were as follows: allergy to local anesthetics; infection at the puncture site or inability to distinguish anatomical structures under ultrasonography; cardiovascular symptoms, coagulation disorders, neurological and mental disorders, morbid obesity, significant organ dysfunction, and suspected difficult airways; recent use of sedatives and analgesics; and refusal to provide informed consent. Table 1 presents the baseline characteristics of the patients.

**Table 1:** Baseline characteristics of the patients included in the study

Characteristic	Study group
Age, mean (SD), years	65.43 (3.77)
Height, mean (SD), cm	169.1 (5.85)
Body weight, mean (SD), kg	64.69 (5.96)
Body mass index (SD), kg.m^− 2^	22.61(1.74)
Sex, n (%)	
Women	12 (40.0)
Men	18 (60.0)
ASA grade, n (%)	
I	21 (70)
II	9 (30)
Duration of surgery, mean (SD), min	127.9 (28.32)

ASA, American society of Anesthesiologists; SD, standard deviation

#### Procedure

Venous access was established routinely after entering the operating room. Vital signs were monitored in real time. The following baseline values for the operative side leg were obtained prior to catheter placement in the parasacral ischial plane: maximum voluntary isometric contraction (MVIC) for dorsiflexion at the ankle [9]; assessment of the sensory block using a cold glass vial stored at 5 °C, positioned four finger widths below the fibular head [9]; and simultaneous measurement of the thumb skin and peak systolic velocity (PSV) of the anterior tibial artery (ATA) in both feet.

**Fig. 2:**
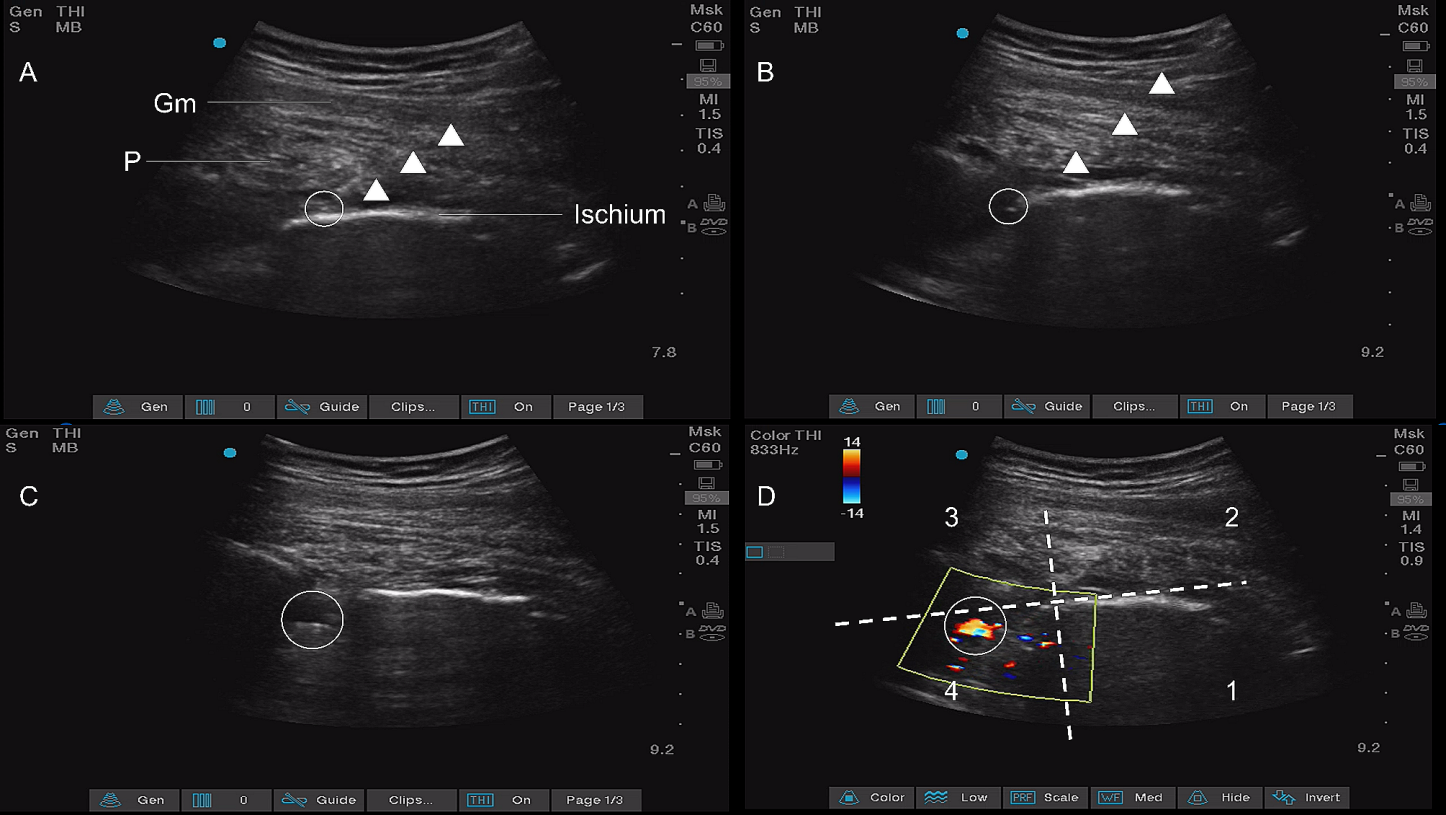
UGCPIPB (**A**) The needle tip contacts the posterior border of the sciatic bone, and water separates the plane between the fascia over the piriformis and the ischium. (**C**) The puncture needle enters along the posteromedial border of the ischium into the deep side of the greater sciatic foramen. (**C**) The catheter is inserted through the needle. (**D**) The catheter tip was located in the fourth quadrant.The white triangle represents the needle’s trajectory. Gm, Gluteus maximus; P, Piriformis

An experienced anesthesiologist performed all blocks, and the patients were placed on the non-operative side in the lateral position. A 2–5 MHz curvilinear probe was positioned along the line connecting the posterior superior iliac spine (PSIS) and greater trochanter, with the medial edge of the probe aligned directly on the PSIS. The probe was subsequently shifted using the parasacral parallel shift (PSPS) approach, moving inferomedially. The piriformis was found to be covering the posteromedial border of the ischium, which was located at the level of the greater sciatic foramen. The skin was disinfected with 75% alcohol. Local infiltration anesthesia was induced using infiltration lidocaine. A 1.2 × 120 mm needle (Baiyue Medical Technologies, AN-NI, China) was inserted using an in-plane approach from the lateral side toward the posteromedial border of the ischium. Following bony contact, 4 mL normal saline was injected, with careful observation of the appropriate hydro dissection of the characteristic fascial plane (Fig. 2A). The direction of the puncture needle was adjusted to enter along the posteromedial border of the ischium into the deep side of the greater sciatic foramen (Fig. 2B). Ultrasound Doppler mode was used to position the frame on the deep side of the greater sciatic foramen. Three milliliters of normal saline were injected through the puncture needle to confirm that the needle tip was beneath the fascia over the piriformis. Thereafter, an anesthetist inserted the catheter using ultrasound guidance alone to a maximum distance of 4 cm beyond the needle tip (Fig. 2C) [10].

The catheter was slowly infused with 2 mL of normal saline to observe the position of the catheter tip. For the convenience of follow-up, we used two lines to divide the ultrasound image into four quadrants, with the posteromedial border of the ischium as the central point. We referred to the parallel line, located between the ventral side of the fascia over the piriformis and the posteromedial border of the ischium, as LINE A, and the vertical line, drawn from the posteromedial border of the ischium, as LINE B (Fig. 2D). The catheter tip was in the fourth quadrant, below the piriformis and posterior medial border of the ischium, thereby indicating the correct positioning of the catheter tip. Following subcutaneous suturing with a tunneled approach and distally ligated (Supplementary Fig. 1A), another confirmation of catheter tip placement was achieved by infusing 3 mL saline solution.

After confirmation (Fig. 2D), 30 mL of 0.5% ropivacaine was administered through the catheter, followed by commercial adhesives (Supplementary Fig. 1B). MVIC was assessed every 5 min after injection of the local anesthetic using a dynamometer (Jamar Smart Digital Hand Dynamometer; Jamar Technology Inc., USA) fixed to a wooden board secured to the bed. The MVIC for dorsiflexion of the anterior tibial muscle was measured in patients lying in the supine position. The MVIC results were averages of three consecutive measurements recorded every min to minimize potential random differences.

#### Outcomes

The primary outcome was to determine the efficacy of initial catheter placement, defined as a reduction of 20% in MVIC for ankle dorsiflexion 30 min after the local anesthetic administration, compared with the baseline measurement [9]. The time to a 20% decrease in MVIC from baseline was defined as the time to motor block onset. Sensory block was scored on a 0–2 scale, where 0, 1, and 2 indicated no anesthesia, hypoesthesia, and complete anesthesia, respectively. This was measured every 5 min, and the time to reach 2 on the scale was defined as the onset time of sensory block [9, 10]. Simultaneously, changes in bilateral blood flow index, lower limb thumb skin temperature, and bilateral PSV of the ATA within 30 min after local anesthetic administration were evaluated [11].

Spinal anesthesia was administered intraoperatively. T_0_ was defined as 6 h after intrathecal administration of ropivacaine. At T_0_, a continuous infusion of ropivacaine 0.2% was initiated from the parasacral ischial plane catheter at a rate of 5 mL.h^− 1^ until T_72_ [12]. The analgesic regimen on the surgical ward included scheduled doses of paracetamol 1 g orally four times a day, ibuprofen 400 mg orally three times a day, and oxycodone 10 mg p.o. every 6 h if VAS > 4/10.

The position of the catheter tip was observed by administering 3 mL of a mixture of 2.5 mL water and 0.5 mL air through the catheter using the above-mentioned ultrasound scanning method [13, 14]. Additionally, five patients required postoperative computed tomography (CT) re-examination. Thus, after obtaining informed consent, we used CT to determine the position of the catheter tip to confirm the accuracy of the previous ultrasound assessment. Postoperative VAS, mobility, bilateral thumb skin temperature of lower limbs, bilateral PSV of ATA, and complications were assessed at 26 °C on postoperative day (POD)1, POD2, and POD3 by the same anesthesiologist during each assessment.

### Stage II

To evaluate the safety and effectiveness of our new technique, we conducted a historical control study contrasting the clinical outcomes of Stage I trial participants with those treated using conventional ultrasound-guided continuous sacral plexus or sciatic nerve blocks via the transgluteal approach. We searched databases, including Web of Science, the Cochrane Library, PubMed, and Embase from the earliest record to November 2023, with a set of keywords (Appendix) for randomized controlled trials that validated the efficacy and safety for ultrasound-guided continuous sacral plexus block or continuous sciatic nerve block in lower limb surgery. We manually reviewed all bibliographies of the included studies for relevant references. We excluded studies that were not written in English or not available in full text.

### Sample size and data analyses

The primary outcome measure was selected based on the established standard deviation (SD) range of 6.3–9.6% for MVIC dorsiflexion in healthy individuals [15]. Therefore, under the assumption of a normal distribution, an MVIC value is inferred to have a 95% probability of falling within the range of the mean ± two SD (mean ± 2SD). Exceeding 2 SDs in MVIC dorsiflexion is considered highly improbable, provided that no other parameters have been altered or affected the MVIC performance (*p* < 0.05). Based on this rationale, we opted to interpret a reduction exceeding two SDs as indicative of a successful block. The primary placement was hypothesized to succeed in ³85% of the cases, necessitating a sample size capable of establishing a lower threshold for the 95% confidence interval (CI) of > 70%. The precision of normal approximation methods for calculating a 95% CI is compromised when dealing with small sample sizes or when the proportion approaches 0 or 1. For a more precise approximation, we used the Wilson interval method for 95% CI estimation [16]. Statistical analyses were performed using GraphPad Prism 8 (GraphPad), and results with *p* < 0.05 were considered significant.

## Results

### Stage I

In a sample of 30 catheter placement cases, the initial placements were successful in 27 cases by injecting a local anesthetic through the catheters, resulting in MVIC reduction of > 20% compared with that at baseline. Three patients did not show a significant decrease (< 20%) in MVIC and were defined as failures. However, the ultrasound assessment confirmed the accurate positioning of the catheter with adequate spread around the sciatic/sacral plexus following the investigators’ initial placement and local anesthetic injection.

**Fig. 3:**
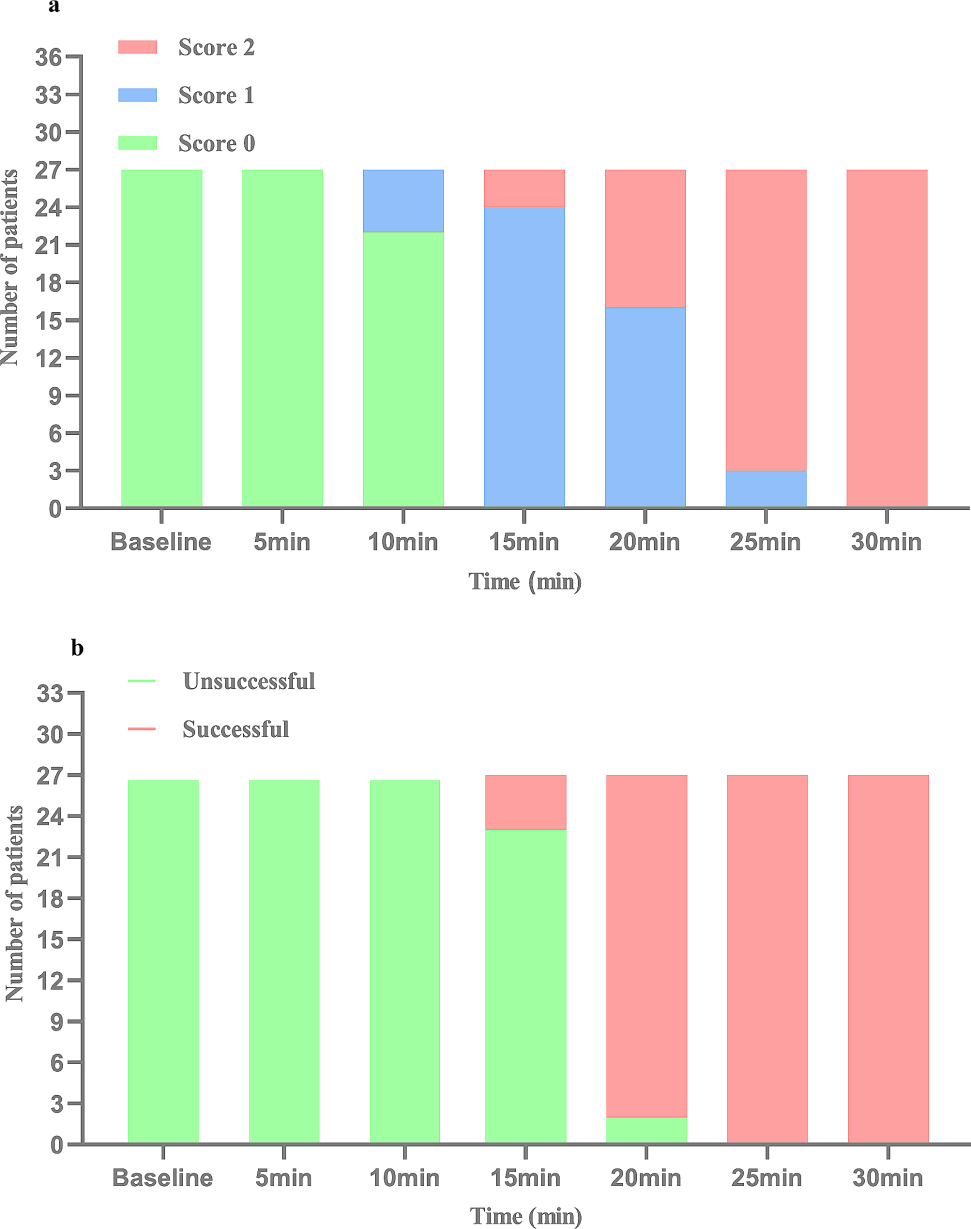
Sensory block and motor block onset time. (**a**) Sensory scores of patients at different time points after ultrasound-guided continuous parasacral ischial plane block. (**b**) The number of patients who achieved maximum voluntary isometric contraction decreased by 20% at different time points after ultrasound-guided continuous parasacral ischial plane block

In the 27 patients with successful initial placements, the MVIC decreased by > 20% at the 25th min in all patients. The onset time of sensory block was a median (range) of 25 (20–25) min, and the onset time of motor block was a median (range) of 20 (20–20) min in these 27 patients. Sensory and motor block onset times are shown in Fig. 3.

**Fig. 4:**
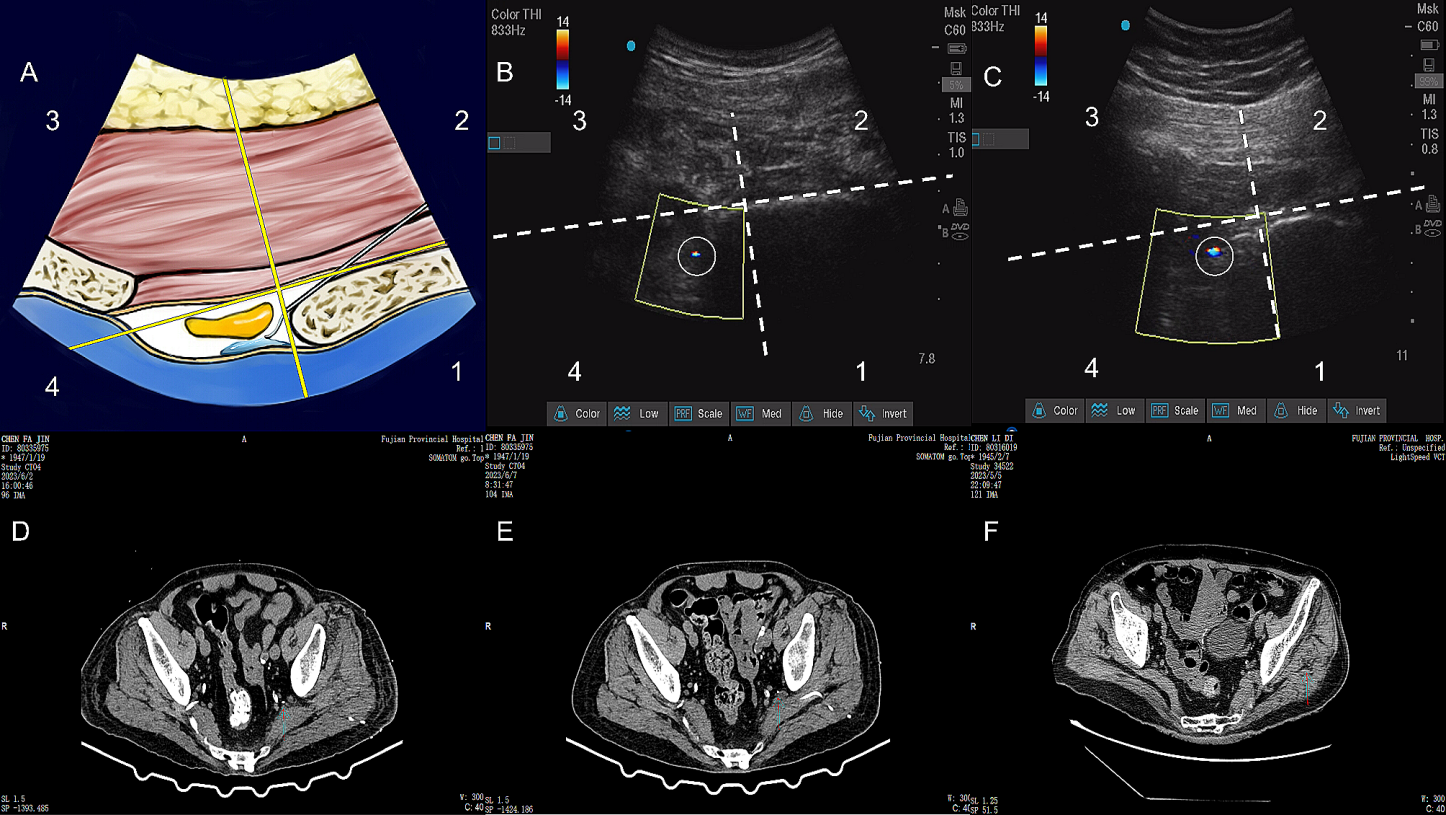
Postoperative catheter tip position assessment (**A**) Schematic of the division of the ultrasound image area. (**B**, **C**) One patient^’^s catheter tip position was assessed using ultrasound on POD1 and POD3. (**D**, **E**) The catheter tip position was assessed in a patient using CT on POD1 and POD5. (**F**) One patient was found to have migration of the catheter tip. The red arrows and white circle both indicate the position of the catheter tip

The catheter tip position for each patient was assessed using ultrasound on POD1–POD3 (Fig. 4A). The catheter tip in the fourth quadrant indicated that the catheter was in place. Catheters were present in 26 patients and displaced in 1 patient. Figure 4B and C show an ultrasound image of the catheter tip in the correct position for a patient on POD1 and POD3, respectively. All catheters were correctly placed on POD1; one patient had catheter displacement with poor analgesia on POD2. Table 2 presents the VAS scores on POD1, POD2, and POD3. Five patients required CT scans after surgery; the results on POD1, POD3, and POD5 in four patients showed that the catheter tip remained in the belly of the fascia over the piriformis (Fig. 4D and E), and the catheter did not show obvious displacement. In one case on POD2, the CT scan revealed that the tip of the catheter had shifted between the gluteus maximus and piriformis, as diagnosed via ultrasound examination (Fig. 4F).

**Table 2:** Opioid consumption, pain scores, mobility, and catheter tip displacement rates

	POD1	POD2	POD3
VAS	3.0 (1.5, 3.5)	2.5 (1.6, 3.2)	2.7 (1.3, 3.4)
Poor analgesia status, n (%)	2 (7.4)	3 (11.1)	3 (11.1)
Times of remedial analgesia	2	4	2
Cumulative OME, mg	30	60	30
Catheter tip displacement, n (%)	0 (0)	1 (3.7)	0 (0)
Ability to stand up, n(%)	25/27 (92.6)	26/27 (96.3)	26/27 (96.3)
Ability to walk, n(%)	22/27 (81.5)	24/27 (88.9)	26/27 (96.3)
Walking > 50 m, n(%)	20/27 (74.1)	25/27 (92.6)	26/27 (96.3)

OME, oral morphine equivalent; POD: postoperative day; VAS: visual analog scale

VAS scores are presented as median (interquartile range); others are presented as number (%)

**Fig. 5:**
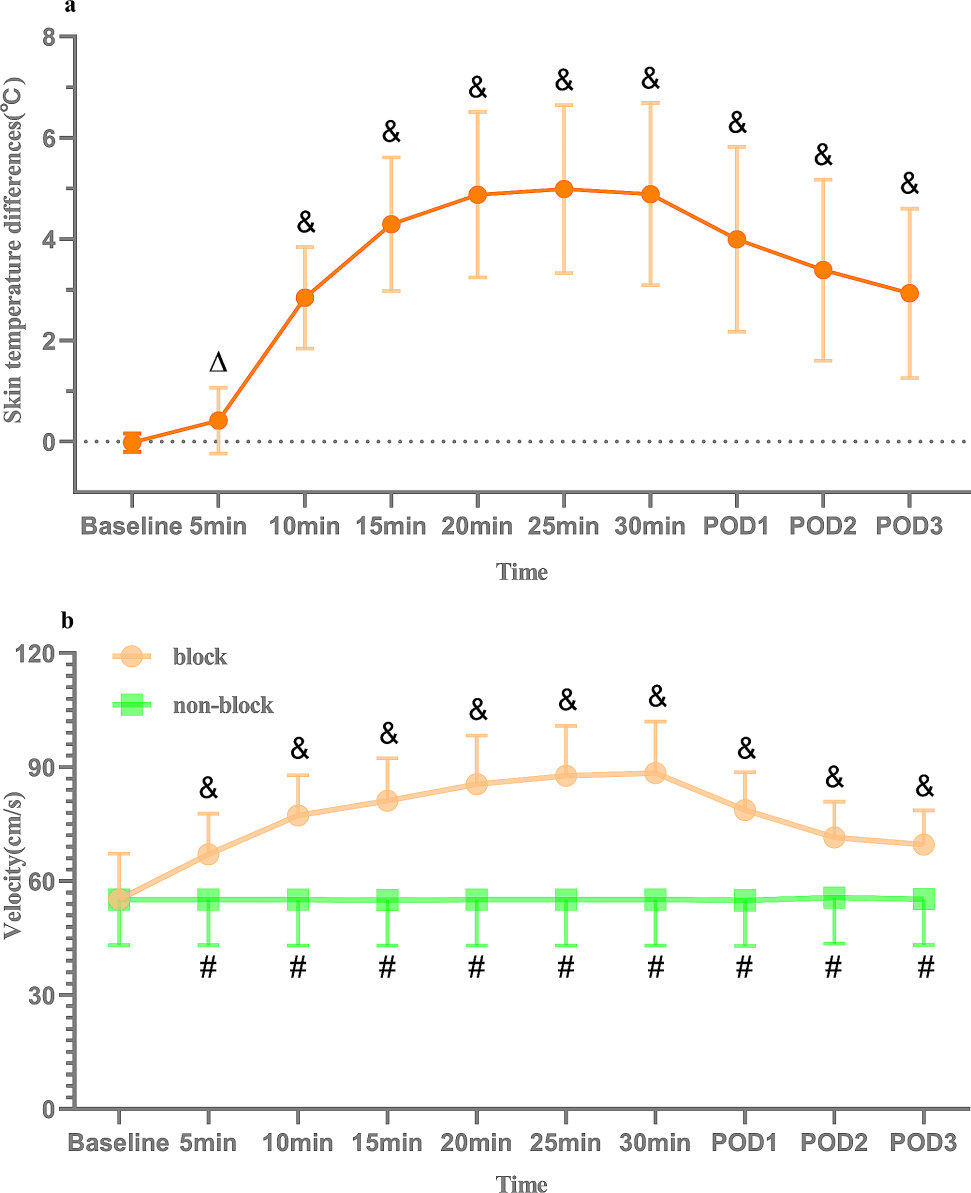
Foot skin temperature differences and PSV of anterior tibial artery (**A**) ∆*p* < 0.01, & *p* < 0.001 (compared with baseline). (**B**) & *p* < 0.001 (compared with baseline), #*p* < 0.001 (block side compared with non-block side)

Skin temperature significantly increased on the blocked side, peaking at 30 min (Fig. 5A). The PSV of the ATA on the blocked side also significantly increased from 30 min post-UGCPIPB to POD3 compared with that on the non-blocked side (*p* < 0.05) (Fig. 5B). Minor complications included catheter clogging in one patient and insertion site leakage in two patients (Table 3).

**Table 3:** Success rate and displacement rate in different studies

	Stage I	Brookes et al. (2015) (10)	*p* -values^a^
Approach	G	G	
Success rate of catheterization, n (%)	(27/30) 90	(31/35) 88.6	1
Rate of catheter dislodgement, n (%)	(1/27) 3.7	(23/96) 24.0	0.012
Complications-catheter clogging, n (%)	(1/27) 3.7	0	0.896
Complications-insertion site leakage, n (%)	(2/27) 7.4	0	0.362
Complications-vascular puncture, n (%)	0	(1/35) 2.9	1
Complications-paresthesia, n (%)	0	(1/35) 2.9	1

^a^All statistics from historical articles were compared with Stage I data and analyzed with Pearson’s chi-square or continuity correction

G, transgluteal approach

### Stage II

A total of 690 relevant articles were identified according to our search strategy; 76 duplicate records were removed, and 562 studies were excluded by title and abstract. Based on the inclusion criteria, 51 studies were excluded after reading the full text. Finally, only one article that contained efficacy data of ultrasound-guided CSNB or CSPB in lower limb surgery was included in our comparative analysis. The new technology showed a high success rate similar to the conventional technology, with no significant difference (*p* > 0.05); however, the rate of catheter displacement with the new procedure was significantly lower than that with the conventional procedure for CSNB (*p* < 0.05).

## Discussion

We demonstrated that UGCPIPB is a simple and effective method for pain management in total knee arthroplasty, indicating that our concept is a promising new therapeutic strategy for total knee arthroplasty. The success rate of UGCPIPB catheter placement reached 90%, similar to that (45–100%) for the first placement of continuous nerve block catheters reported previously [17, 18], but higher than that (88.6%) for sciatic nerve placement using the transgluteal approach [10]. As the criteria for success differed among studies, we selected the more objective MVIC as the criterion for determining the success of catheter placement to avoid subjective bias caused by relying on sensory and motor function scoring to evaluate the effect of the block [9]. The success rate of our new technique may be related to sample size and individual differences. We believe that the success rate could be improved significantly with further practice.

During postoperative continuous block, we observed that patients had relatively effective postoperative analgesia, with a maximum VAS score of 6; poor analgesia was observed in one patient with catheter displacement, similar to the analgesic effect observed in previous studies [19, 20]. By assessing the dermatomal sensory range in patients, we found that UGCPIPB provided effective analgesia for the posterior thigh and knee joint dermatome, posterior knee capsule, calf, ankles, and foot. This finding suggests patients could receive adequate analgesia during lower extremity surgery when combined with continuous nerve blocks, such as FICB and ACB.At the same time, this study also found that UGCPIPB has a minor impact on postoperative knee joint movement and can promote early mobilization of patients.

The presence of a catheter tip in a continuous nerve block is related to the analgesic effect. For the first time, we used the criterion that the catheter tip in the fourth quadrant should be defined as the correct catheter position. This is mainly because when the catheter tip is located between the fascia over piriformis and the presacral fascia, continuous infusion of local anesthetics can effectively wrap the sacral plexus. We found that one patient had catheter displacement on POD2, with a corresponding increase in VAS scores, and a CT examination revealed that the catheter tip appeared between the piriformis and gluteus maximus, which is consistent with our ultrasonic scan results. This finding suggests using ultrasonic Doppler mode to determine if the catheter tip is in place. Our study revealed a catheter displacement rate of 3.7%, which is lower than that for conventional continuous sciatic block in Stage II; this may be related to the trajectory of our catheter and parasacral ischial plane. Theoretically, various parameters can influence catheter displacement, including catheter design, catheter placement and fixation methods, individual anatomical conditions, or movement of patients. Therefore, further research is needed to elucidate how to reduce the catheter displacement rate of UGCPIPB.

The increase in skin temperature at the corresponding innervation site after nerve block often indicates the success of nerve block [11]. Here, after successful placement of the catheter in the parasacral ischial plane, administration of 30 mL 0.5% ropivacaine resulted in a median time of 10 (10–10) min for a 2 °C increase in temperature on the non-blocked side of the lower limb [21], with a maximum temperature difference observed at 25 min, averaging 5.0 ± 1.7 ℃. During POD1–POD3, the average temperature increase, compared with that on the non-blocked side, was 3.4 °C ± 1.7 °C. Similar to the increase in skin temperature, PSV in the lower limbs is an objective indicator of the success of a nerve block [22]. Here, the PSV of the ATA on the blocked side was significantly higher than that on the non-blocked side. The increase in skin blood flow index, temperature, and PSV after nerve block is considered caused by the blockade of sympathetic nerve fibers or sympathetic ganglia [23, 24]. An anatomical study of the parasacral ischial plane found that the sacral sympathetic trunk and sacral plexus were located within the fascia over the piriformis and presacral fascia planes. Diffusion of a local anesthetic in this plane is likely to block the sacral sympathetic trunk [25, 26]. Blocking the sacral sympathetic trunk can result in a more precise increase in lower-limb blood flow velocity and skin temperature, making this technology applicable to lower-limb anesthesia and analgesia and treating vasospastic pain diseases of the lower limbs.

The incidence of complications associated with UGCPIPB was relatively low. The sacral plexus nerve block or sciatic nerve block via the transgluteal approach is not widely applied in clinical practice because of technical difficulties compared to other methods and concerns regarding the complications of deep nerve blocks. By improving the conventional continuous sacral plexus block by utilizing UGCPIPB, we found a low probability of nerve and vascular injuries. However, during the puncture process, tracing the sciatic nerve route and identifying and avoiding the superior gluteal artery via the ultrasonic Doppler mode are extremely important. During UGCPIPB, we did not detect any infection around the catheter; however, aseptic block operation and postoperative catheter care are extremely important. Additionally, our study did not find urinary incontinence complications, which are a concern during the sacral plexus block. This is likely because anesthetics did not block the inferior hypogastric plexus ascending along the parasacral ischial plane, which primarily innervates the detrusor muscle of the bladder during the micturition reflex [26–28].

Compared to previous studies in Stage II, studies on ultrasound-guided continuous sacral plexus block and sciatic nerve block via the transgluteal approach are limited, possibly due to the technical challenges associated with this technique. Continuous sacral plexus block or sciatic nerve block via the gluteal approach are often guided by nerve stimulators, leading to multiple punctures and uncertain effects. Therefore, through our technical improvement, we can perform ultrasound-guided continuous sacral plexus block more conveniently and effectively, and only continuous sacral plexus block can provide analgesia for the lower limb, such as the hip joint or above the knee joint.

This study had some limitations. First, this proof-of-concept study had a small sample. For such a novel lower limb analgesia technology, a preliminary feasibility study is necessary, and a randomized controlled trial with a large sample is required to verify the analgesic and anesthetic treatment effects of this technology. Second, although this is the first study to propose a standardized UGCPIPB catheterization procedure, with preliminary observations showing a relatively low catheter displacement rate on POD3, studies with larger samples and longer observation durations are necessary to assess long-term catheter displacement rate, leakage rate, and safety of prolonged local anesthetic infusion. Third, catheter displacement was assessed using postoperative ultrasound imaging, thereby considering the plane in which the greater sciatic foramen divides the belly of the fascia over piriformis and posteromedial border of the ischium into four quadrants. This method can only ensure that the catheter tip is in a relatively correct anatomical position. Subsequently, the catheter tip position can be further determined by incorporating sensory and motor assessments. Fourth, because this catheterization technique is based on UGCPIPB, we had to use normal saline to expand the plane between the fascia over the piriformis and ischium, thereby allowing the catheter to travel within the fascial plane. Although we controlled the amount of normal saline injected during the catheterization process, excessive use of normal saline may dilute the local anesthetic solution, resulting in delayed effectiveness or blockade failure.

In conclusion, UGCPIPB appears to be a safe and effective method for achieving continuous sacral plexus block associated with total knee arthroplasty analgesia. This procedure has the potential to improve lower limb analgesia approaches.

### Electronic supplementary material

Below is the link to the electronic supplementary material.

Supplementary Material 1

## Data Availability

Data are available depending on the request.

## Appendix

A literature search was made using the following terms: ((“continuous nerve block” OR “continuous nerve blockade” OR “continuous regional anaesthesia” OR “continuous analgesia” OR “continuous anaesthesia” OR “continuous peripheral nerve block” OR “continuous peripheral nerve blockade” OR “continuous peripheral nerve catheters” OR “peripheral nerve block” OR “peripheral nerve blockade” OR “peripheral nerve catheter” OR “peripheral nerve catheter placement” OR “perineural catheter” OR “perineural catheter insertion” OR “perineural catheter placement” OR “perineural catheter technique” OR “nerve catheters”) AND (“lower limb” OR “knee” OR “ankle” OR “foot” OR “calcaneal” OR “femoral” OR “tibia” OR “fibula”)) as found in the title or abstract under the Medical Subject Heading (MeSH) “Anaesthesia and Analgesia.”

## Abbreviations

ACB: Adductor canal block
ATA: Anterior tibial artery
BMI: Body mass index
CT: Computed tomography
CPNB: Continuous peripheral nerve block technique
CSPB: Conventional continuous sacral plexus block
CSNB: Continuous sciatic nerve block
UGCPIPB: Ultrasound-guided continuous parasacral ischial plane block
FICB: Fascia iliaca compartment block
MVIC: Maximum voluntary isometric contraction
POD: Postoperative days
PSIS: Posterior superior iliac spine
PSPS: Parasacral parallel shift
PSV: Peak systolic velocity
PIPB: Parasacral ischial plane block
VAS: Visual analog scale
