# Supplementary figures and images for: A proof-of-concept study of ultrasound-guided continuous parasacral ischial plane block for postoperative pain control in patients undergoing total knee arthroplasty
Source: J Orthop Surg Res. 2024 Jun 8;19:339. doi: 10.1186/s13018-024-04822-9 (PMC11162005; doi:10.1186/s13018-024-04822-9)

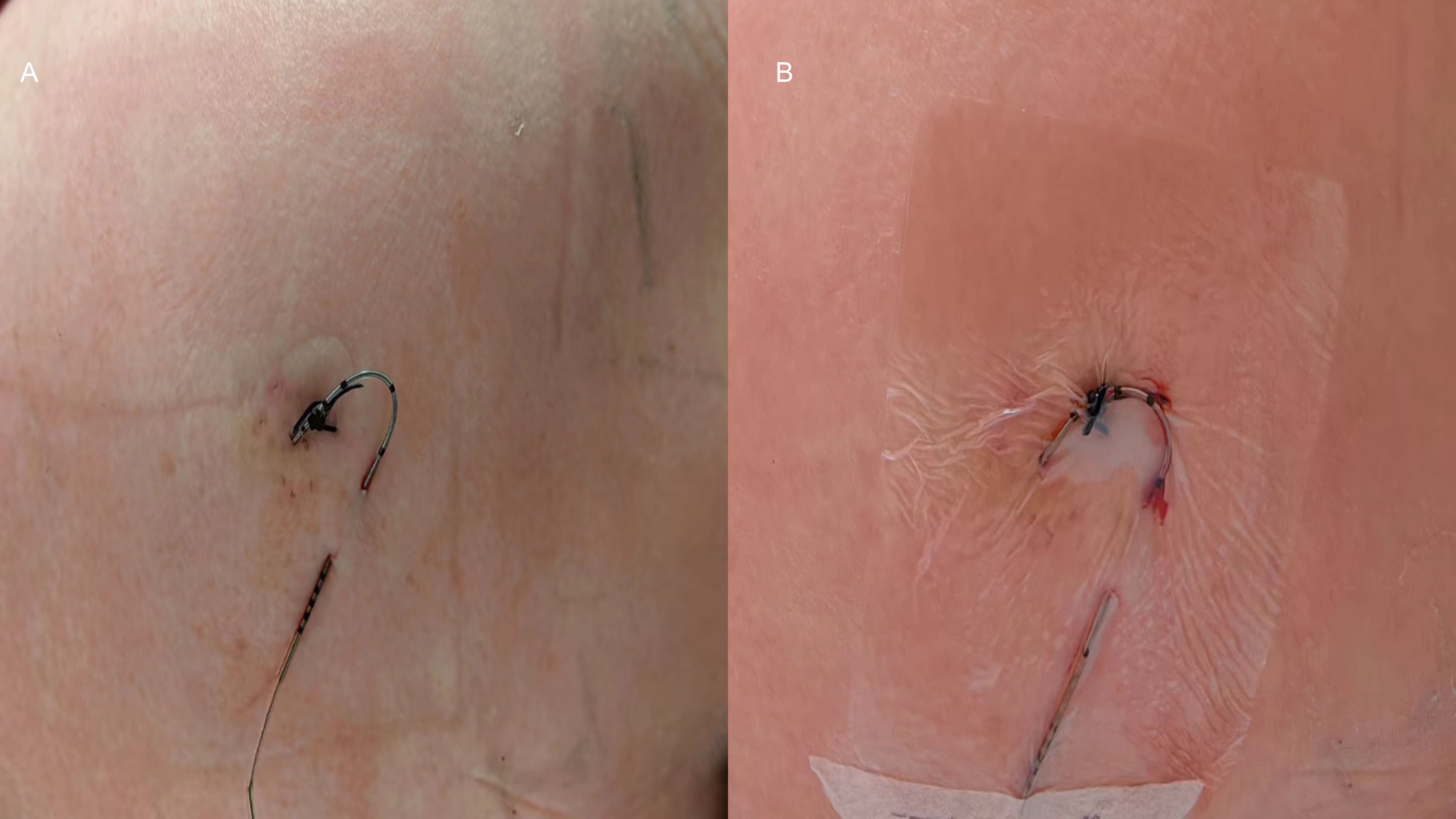

Supplement: Supplementary file 1 — Supplementary Material 1 [file 13018_2024_4822_MOESM1_ESM.tif]
